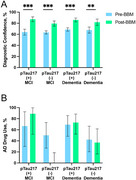# Clinical Utility of Plasma pTau217 and NfL in Dementia Diagnosis and Management in a Taiwanese Memory Clinic

**DOI:** 10.1002/alz70856_106176

**Published:** 2026-01-08

**Authors:** Yu‐Wen Cheng, Ta‐Fu Chen

**Affiliations:** ^1^ Department of Neurology, National Taiwan University Hospital, Taipei, Taiwan

## Abstract

**Background:**

Blood‐based biomarkers (BBMs) are emerging as promising tools for the biological diagnosis of dementia. However, studies examining the clinical relevance of BBMs in Asian populations remain limited. This study aimed to explore the impact of plasma phosphorylated tau (pTau) and neurofilament light chain (NfL) on clinical diagnosis and management decisions in a memory clinic cohort in Taiwan.

**Methods:**

Patients with mild cognitive impairment (MCI) or dementia, with diagnostic confidence below 80%, were recruited from the National Taiwan University Hospital memory clinic between November 2023 and July 2024, prior to the clinical availability of anti‐amyloid monoclonal antibodies in Taiwan. Plasma pTau217 and NfL levels were measured using the ALZpath Simoa assay. Plasma pTau217 levels were categorized using a three‐range approach, with the intermediate zone defined as 0.40–0.63 pg/mL and levels above 0.63 pg/mL indicating AD pathology. Clinical diagnoses were categorized as AD, non‐AD degeneration, or non‐neurodegenerative processes. Diagnoses, diagnostic confidence, and management plans were assessed before and within 90 days after plasma biomarker measurement. Changes in diagnosis, diagnostic confidence, and AD drug therapy were compared before and after the application of BBMs.

**Results:**

Seventy‐two patients (mean age 72.5 ± 8.4, 40% women, 37.5% with MCI) were enrolled for BBM measures. Elevated plasm pTau217 levels were observed in 9 (33%) MCI and 27 (60%) dementia patients. Disclosure of plasma pTau217 and NfL levels was associated with changes in etiology diagnosis in 11 (41%, 95% CI= 21‐61%) MCI and 10 (22%, 95% CI=0.2‐18%) dementia patients. Additionally, changes in AD drug therapy occurred in 11 (41%, 95% CI 21–61%) MCI and 4 (9%, 95% CI 0.2–18%) dementia patients. Diagnostic confidence improved from 64% to 82% for MCI patients and from 68% to 84% for dementia patients. For anti‐amyloid antibody therapy eligibility, 7 (30%) of the 23 potentially eligible participants could avoid an amyloid PET study due to low pTau217 level. Two patients previously diagnosed with non‐AD degeneration became eligible due to elevated pTau217 levels.

**Conclusions:**

Plasma pTau217 and NfL biomarkers may significantly influence clinical diagnosis and management decisions in patients with MCI and dementia.